# Palmitoylethanolamide and Related ALIAmides: Prohomeostatic Lipid Compounds for Animal Health and Wellbeing

**DOI:** 10.3390/vetsci7020078

**Published:** 2020-06-16

**Authors:** Enrico Gugliandolo, Alessio Filippo Peritore, Cristian Piras, Salvatore Cuzzocrea, Rosalia Crupi

**Affiliations:** 1 Department of Chemical Biological Pharmaceutical and Environmental Sciences, University of Messina, 98168 Messina, Italy; egugliandolo@unime.it (E.G.); aperitore@unime.it (A.F.P.); 2 Department of Chemistry, Reading University, Reading RG6DX, UK; c.piras@reading.ac.uk; 3 Department of Pharmacological and Physiological Science, Saint Louis University School of Medicine, St. Louis, MO 63104, USA; 4 Department of Veterinary Science, University of Messina, 8168 Messina, Italy; rcrupi@unime.it

**Keywords:** palmitoylethanolamide PEA, ALIAmides, autacoid local injury antagonism, endocannabinoid, wellbeing

## Abstract

Virtually every cellular process is affected by diet and this represents the foundation of dietary management to a variety of small animal disorders. Special attention is currently being paid to a family of naturally occurring lipid amides acting through the so-called autacoid local injury antagonism, i.e., the ALIA mechanism. The parent molecule of ALIAmides, palmitoyl ethanolamide (PEA), has being known since the 1950s as a nutritional factor with protective properties. Since then, PEA has been isolated from a variety of plant and animal food sources and its proresolving function in the mammalian body has been increasingly investigated. The discovery of the close interconnection between ALIAmides and the endocannabinoid system has greatly stimulated research efforts in this field. The multitarget and highly redundant mechanisms through which PEA exerts prohomeostatic functions fully breaks with the classical pharmacology view of “one drug, one target, one disease”, opening a new era in the management of animals’ health, i.e., an according-to-nature biomodulation of body responses to different stimuli and injury. The present review focuses on the direct and indirect endocannabinoid receptor agonism by PEA and its analogues and also targets the main findings from experimental and clinical studies on ALIAmides in animal health and wellbeing.

## 1. Nutrition-Oriented Health Promotion in Animals

Nutrition is generally regarded as the process of taking in food and using it for growth, metabolism, and repair [1]. Virtually every cellular process is affected by diet and lifestyle. Food components interact with cell metabolic functions accordingly. Similar to drugs, dietary compounds act as modifiers of network function and stability [2,3]. This represents the foundation of dietary management, which is currently and successfully applied to a variety of clinical conditions of dogs and cats [1]. The nutritional approach to pet health feed is nowadays considered a very promising field, from a clinical and marketing perspective. Based on recent estimates, the feed-related industry totals more than $250 billion per year, and the feed-based approach to animal health and diseases is more popular than in humans [4]. Several naturally occurring compounds are regularly used as feed materials worldwide and they are under Regulation (EC) No 767/2009 in European countries. They are present in food sources (although not all of them are normal constituents of the mammalian body) and have repeatedly been shown to exert anti-inflammatory and pain-relieving effects in several experimental studies and clinical trials in veterinary patients affected with various disorders, especially of a chronic nature [4]. Glucosamine, omega-3 and -6 essential fatty acids, and methylsulfonylmethane are just few examples. There is substantial evidence that polyunsaturated fatty acids from vegetable and fish oil (e.g., eicosapentaenoic, docosahexaenoic, and linoleic acid) exert important benefits in a range of veterinary diseases, from atopic dermatitis [5,6,7] to renal insufficiency [8,9] and cognitive dysfunction syndrome [10]. Moreover, omega-3 essential fatty acids have been widely and successfully applied to osteoarthritis [11,12,13,14,15], similarly to glucosamine [16,17,18,19] and the organosulfur compound methylsulfonylmethane [4]. Thus, the interaction between illness, health, and nutritional status is multifactorial and complex. Several health benefits have been shown for nearly any feed material, in line with the famous quote generally attributed to Hippocrates “Let food be thy medicine, and let medicine be thy food.” For a large number of compounds used in the so-called nutraceutical approach to health, data on safety and toxicity are still missing and pharmacokinetic, pharmacodynamic, and toxicological studies are few, if any [4]. This is not the case for the naturally occurring compound palmitoylethanolamide (PEA) and related amides, which are being increasingly investigated in recent years, as regarding health-promoting features and safety issues. The present review will examine and discuss the food sources, physiological role, and the most updated data on the bioavailability and benefits of PEA and congeners, with special reference to the effect on animal health.

## 2. Natural Presence of PEA in Vegetable and Animal Food Sources

In 1950, it was discovered that less privileged children fed with an egg-rich diet were apparently protected against rheumatic fever [20]. The protective factor was found to be a lipid fraction from the egg yolk (which was also identified in peanut oil and soybean lecithin) and discovered to be a particular lipid amide known as palmitoyl ethanolamide, PEA [21,22] The protective properties of PEA led many researchers to investigate its presence in other natural sources. PEA was thus found in the seeds of some varieties of legumes, such as peas and beans [23,24], as well as green/roasted coffee and cocoa [25,26,27]. Moreover, many other food sources of PEA were progressively discovered, like, for example, tomatoes, alfalfa (*Medicago sativa*), potatoes, carrots, walnuts, peanuts, wheat flour, barley, tuna fish, and vegetable oils [23,24,25,26]. Moreover, high levels of PEA were also found in human, bovine, and elk milk [25,28,29,30] (Table 1). Interestingly, a high and increasing amount of PEA has recently been found in milk samples from mothers belonging to underserved populations, where milk represents a food of primary importance to avoid infant malnutrition [31]. Accordingly, deregulated levels of PEA in breast milk were repeatedly found to negatively affect the growth of offspring [32,33]. One might thus speculate that PEA represents an early nutrient. The presence of PEA is not limited to natural foods; indeed, physiological PEA levels were also detected in virtually any tissue and body fluid. In particular, PEA is produced in the muscle and spleen but also the eyes, gastrointestinal tract, spinal cord, skin, heart, blood, and subcutaneous adipose tissue [34,35,36,37,38,39]. Moreover, PEA levels are also abundant in the brain from different species, such as pigs, sheep, cows, guinea pigs, mice, and rats [34,40,41,42,43,44,45,46,47,48]. In addition, PEA has also been quantified in reproductive fluids, such as seminal plasma, follicular, oviductal, and amniotic fluid, as well as the umbilical vein and artery, the main nutritional sources of the fetus [28,29,49]. Finally, human and canine synovial fluid also contains noticeable levels of PEA [50,51]. This vast distribution of PEA is nowadays viewed as a crucial prerequisite for its main function, namely endogenous protection in response to different types of damage.

## 3. ALIAmides and the Highly Conserved N-acylethanolamine Family

**Figure 1:**
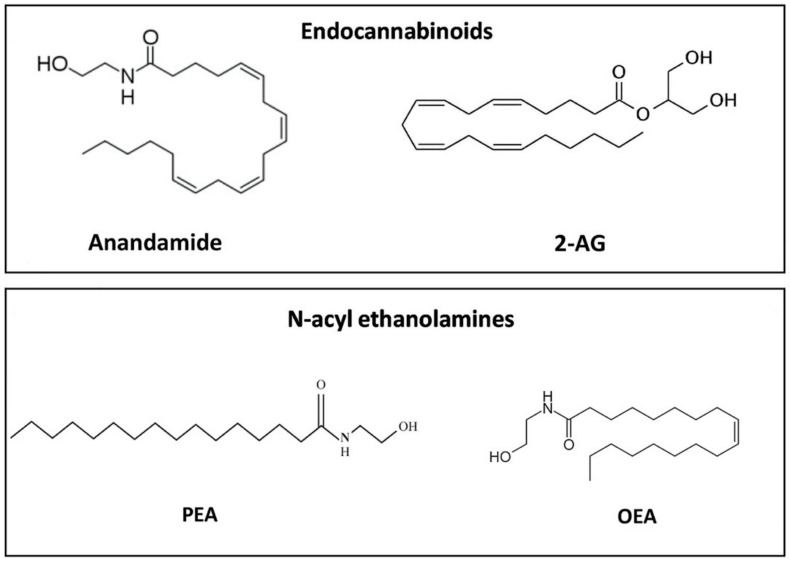
Chemical structure of representative endocannabinoids and related N-acyl ethanolamines.

The endogenous amides, acting through the autacoid local injury antagonism mechanism (ALIA mechanism), are a class of naturally occurring molecules, i.e., ALIAmides, named for the first time in 1993 by the late Nobel prize winner Rita Levi Montalcini [52]. The term “autacoids” comes from the Greek "autos" (self) and "acos" (healing or remedy) and refers to cell-produced factors that act locally, i.e., near their site of synthesis [53]. Originally considered as mediators of inflammation (e.g., histamine), autacoids were soon discovered not only to induce but also to reduce inflammation and, more generally, tissue injury [53]. Since then, the possible prohomeostatic role of autacoids has aroused great scientific interest and has been increasingly interwoven with the research field involved in the resolution of inflammation, or more simply resolution [54]. The autacoid ALIAmides are endogenous bioactive N-acyl ethanolamines (NAEs), which regulate a variety of physiological functions and are biosynthesized in response to cellular stress and tissue damage with prohomeostatic purposes, i.e., to balance the internal environment in both plants [55] and animals [56]. NAEs are considered an evolutionarily conserved lipid signaling system. The machinery for the biosynthesis and degradation of NAEs is functionally conserved in both animal [57,58] and plant systems [59]. There are numerous endogenous NAEs, like oleoyl ethanolamide (OEA), stearoyl ethanolamide (SEA), and other less studied molecules, such as eicosatrienoyl ethanolamide (ETEA). The most researched and well-known among them are the endocannabinoid arachidonoyl ethanolamide (AEA or anandamide) and the endocannabinoid-like PEA, the parent molecule of ALIAmides (Figure 1). PEA is one of the most highly conserved NAEs during evolution and it is found even in microscopic single-cell organisms, like the yeast *Saccharomyces cerevisiae* [60] as well as invertebrates (e.g., mollusks) [61]. Evolutionary conservation underscores the functional importance of NAEs, and particularly PEA. Recently, some studies have highlighted the benefits of newly developed molecules acting through the so-called ALIA mechanism, like Adelmidrol, a derivative of azelaic acid, and N-palmitoyl-D-glucosamine or Glupamid. Some relevant findings will be briefly presented in the last part of this review.

## 4. Endogenous PEA: Metabolic Pathways and Change in Tissue Levels

**Figure 2:**
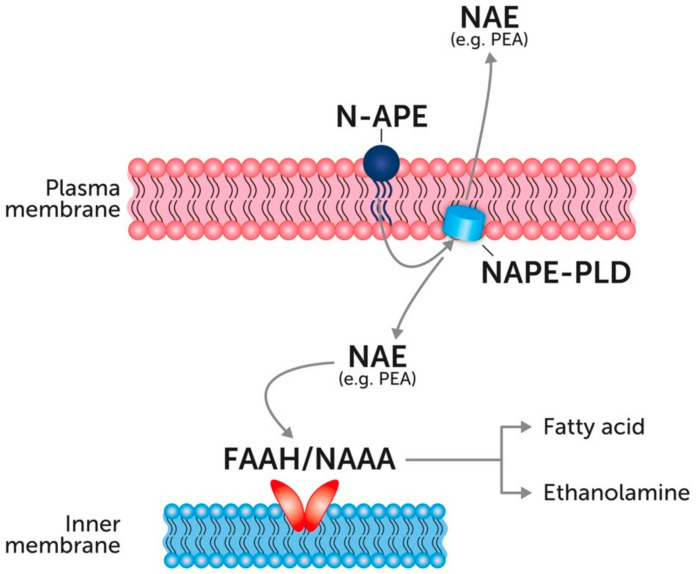
The main biosynthetic and degradative pathways of N-acyl ethanolamines (NAEs). Modified from [56]. NAE: N-acyl ethanolamines; NAPE-PLD: N-acyl phosphatidylethanolamine phospholipase D; NAPE: N-acyl phosphatidylethanolamine; FAAH: fatty acid amide hydrolase; NAAA: N-Acylethanolamine acid amidase

PEA, like other NAEs and related endocannabinoids, is synthesized and metabolized by different animal cell types and also present in plants, as recently reviewed by Petrosino and Di Marzo [62]. NAEs are biosynthesized from atypical phospholipids of cell membranes bearing three acyl chains (i.e., N-acyl-phosphatidyl ethanolamines, NAPEs), with the synthesis involving two enzymes, i.e., NAPE-generating Ca2+-dependent N-acyltransferase (Ca-NAT) and NAPE-hydrolyzing phospholipase D (NAPE-PLD) [63]. Like the other NAEs, PEA is produced "on demand" by most cells and its local levels are strictly regulated by balancing the activity of the biosynthetic and degradative metabolic pathways. The primary degrading enzymes are fatty acid amide hydrolase (FAAH) and N-acyl ethanolamine-hydrolyzing acid amidase (NAAA) [64,65] (Figure 2). Interestingly, PEA levels have been shown to change during stressful conditions. The first observation in this regard came from a study performed in dogs. It was found that the infarcted areas of canine myocardium contained substantial amounts of NAEs (about 20-fold higher than normal heart muscle), the main one being PEA. It was thus speculated that PEA was produced as a response to ischemic injury and might exert beneficial effects in the infarcted area [35]. Since then, several studies have characterized the changes in PEA levels during different pathophysiological conditions [66,67,68]. For example, it has been shown that epidermal cells subjected to UV irradiation, which is known to induce cell damage, produce considerable amounts of NAEs, with PEA showing the highest increase [69]. Furthermore, in the lesional skin of privately owned dogs affected with atopic dermatitis, the levels of NAEs as well as those of the classic endocannabinoid, 2-arachydonoylglycerol (2-AG), were shown to be significantly elevated compared to normal non-atopic skin, with PEA levels showing the highest increase (more than 30 fold) [36]. A great deal of evidence suggests that PEA metabolism (i.e., the overall set of endogenous biosynthetic and degradative pathways) may be disturbed during certain disorders [70]. Accordingly, tissue levels of PEA are increased in several disease conditions and a decrease in PEA levels contributes to the disease development [71,72,73]. The most commonly accepted hypothesis is that the synthesis of PEA increases when tissues face an actual or potential injury and serves as an early stop signal that contrasts the progress of inflammation [74]. Accordingly, one might argue that pathological situations may arise in which endogenous PEA levels are inadequate in dealing with the ensuing insult. In these cases, exogenous administration to effectively ‘top up’ the body’s own supply may be a viable approach [56].

## 5. PEA Mechanism of Action: A Multitarget Redundancy

**Figure 3:**
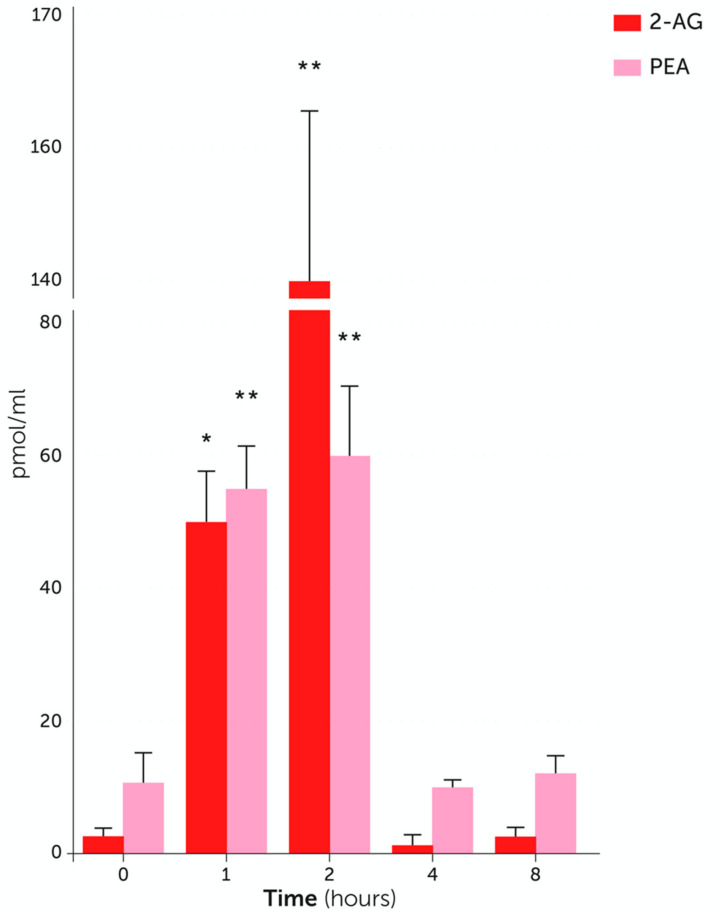
PEA (palmitoyl ethanolamide) entourage effect on 2-AG (2-arachydonoylglycerol). Following a single dietary supplementation with PEA-um to hypersensitive Beagle dogs, 2-AG plasma levels are significantly elevated, with a slight delay compared to PEA ones. * *p* < 0.05 and ** *p* < 0.001 versus time 0. Modified from [87].

In addition to the beneficial effects and metabolic pathways, several studies have focused on the mechanisms of action of PEA. The key result is that PEA acts through multiple pathways, both at the cellular and molecular level (recently reviewed in [62]). In regard to cellular targets, it was originally observed that PEA downmodulates mast cell behavior after challenge, i.e., the ALIA mechanism [53]. The finding was later confirmed in dogs and cats, with PEA being able to control mast cell releasability in different settings, e.g., freshly isolated cells from canine skin biopsies [75], canine skin organ cultures [76], hypersensitive dogs [77], and cats affected by eosinophilic granuloma and eosinophilic plaque [78]. Besides mast cells, different cell populations were also shown to be targets of PEA. Indeed, once activated, macrophages, keratinocytes, T cells, astrocytes, and microglia are all negatively controlled by PEA [71,79,80,81,82,83,84]. From the molecular side, PEA appears to act through multiple receptors, some behaving as direct while others as indirect targets. In particular, PEA can directly activate PPAR-α (peroxisome proliferator-activated receptor α) [85] or, more controversially, GPR55 (G-protein-coupled receptor 55) [86]. On the other hand, the activation of canonical cannabinoid receptors (i.e., Cannabinoid receptor (CB) type 1 CB1 and CB2) depends on the PEA-induced increase of endocannabinoids, like AEA or 2-AG [87,88,89]. The term “entourage effect” was coined to explain the aforementioned indirect effect of PEA on cannabinoid receptors, through the increased availability of endocannabinoid(s) [89,90,91]. Interestingly, the entourage effect was specifically shown in dogs, with oral administration of PEA in its bioavailable form (i.e., ultra-micronized, see below) being paralleled by a significant and up to ~20-fold increase in the plasma levels of 2-AG [87] (Figure 3). The entourage effect seems also to guide, at least in part, the interrelation between PEA and transient receptor potential vanilloid 1 (TRPV1), whose activation and desensitization depends on the PEA-induced AEA or 2-AG increase [87,89,90,91]. To make it even more complex, PEA’s action on PPAR-α is also responsible for increasing CB2 expression and TRPV1 activation [92,93].

It is now clear that PEA is an endocannabinoid-related compound, sharing metabolic pathways and targets with the endocannabinoid system, which is now considered to play an integral role in maintaining body homeostasis [94]. Most notably, the heterogeneous family of canonical and putative cannabinoid receptors, i.e., PEA targets, are being extensively studied in companion animals and their distribution has been found in several body tissues [95,96,97,98,99,100,101,102,103,104,105,106,107,108,109]. Table 2 provides a summary of the main results.

## 6. PEA as A “Proresolving” Lipid Mediator

**Figure 4:**
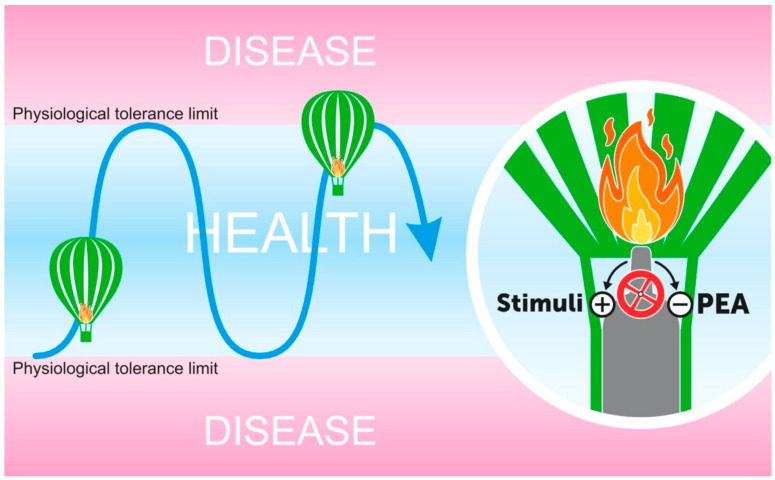
The prohomeostatic function of PEA: A conceptual view. Just like a hot air balloon floating in the sky, tissue homeostasis requires persistent monitoring and adjustments as conditions change. If the flame from the burner is too high (+), the rising balloon might overcome the upper safety limits. On the contrary, if the air inside the balloon is too cool, the opposite might happen. PEA helps to finely tune the “burner knob” and counterbalances the excessive burst, and related tissue hyper-reactivity, in response to high-intensity stimuli (-)**.**

Inflammation is a natural body response to harmful stimuli (e.g., pathogens, irritants) serving protective purposes. It is generally believed that a controlled inflammatory response is beneficial, but it can become detrimental if dysregulated, with uncontrolled inflammation being a key player in the pathogenesis of several diseases [110,111]. Therefore, inflammation must be finely tuned and switched off when no longer needed [112]. Accordingly, proinflammatory mediators, such as arachidonic acid derivatives, cytokines, and chemokines, should be finely counterbalanced by the so-called “proresolving” mediators, in order to limit inflammation and terminate the response once the threat has passed [113,114,115]. It can be inferred that approaching such a complex process by targeting only one of the biochemical pathways might be a nonoptimal strategy. It is widely recognized that a multitarget approach is more effective, although difficult to achieve with medicinal products, given they usually act in a highly selective way, target a single mechanism, and are generally coupled with side effects as well as drug interactions. An alternative strategy that has been suggested is to commandeer nature’s own anti-inflammatory mechanisms and induce a “dominant” program of resolution [116]. Within this framework, PEA represents an interesting lipid signaling molecule. As outlined in detail in previous chapters, PEA is (i) produced “on demand” in response to tissue damage and/or an inflammatory response; (ii) able to downmodulate cell reactivity, especially at immune-inflammatory cells (i.e., ALIA effect); and (iii) active through a multitarget mechanism involving the prohomeostatic endocannabinoid system [84,117]. In line with this view, dietary supplementation of healthy animals with PEA resulted in a shift in the membrane lipid composition towards a proresolving lipid environment [118]. Therefore, dietary supplementation of PEA and congeners substantially overcomes the classical pharmacology view of “one drug, one target, one disease”, opening a whole new era in the management of animals’ health, i.e., an according-to-nature biomodulation of body responses to different stimuli and injury. A conceptual view of the prohomeostatic function of PEA is shown in Figure 4.

## 7. PEA Bioavailability: A Size Issue

**Figure 5:**
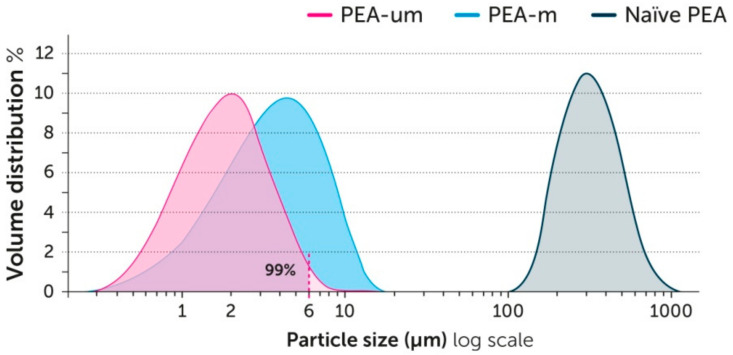
Particle size distribution profile of different PEA formulations. More than 99% and about 60% by weight of PEA-um has a particle size lower than 6 and 2 μm, respectively.

The health-promoting effect of any dietetic compound can be limited because of lipophilicity and intrinsic low dissolution rate(s). Both features will necessarily translate into scarce absorption, and poor pharmacokinetics and bioavailability [119]. PEA is practically insoluble in water and scarcely soluble in most aqueous solvents, with the logarithm of its partition coefficient (log P) being >5 [120]. Since the absorption of lipophilic substances is inversely proportional to the size of their particles [121], one of the most suitable systems for optimizing the functions of PEA following oral administration is to use a microgrinding process, called micronization. As will be discussed below, the oral administration of micronized PEA, and more particularly that with a particle size between 0.6 and 6 μm (the so-called PEA-um or ultra-micronized PEA, Figure 5), shows superior activity compared to other forms.

**Figure 6:**
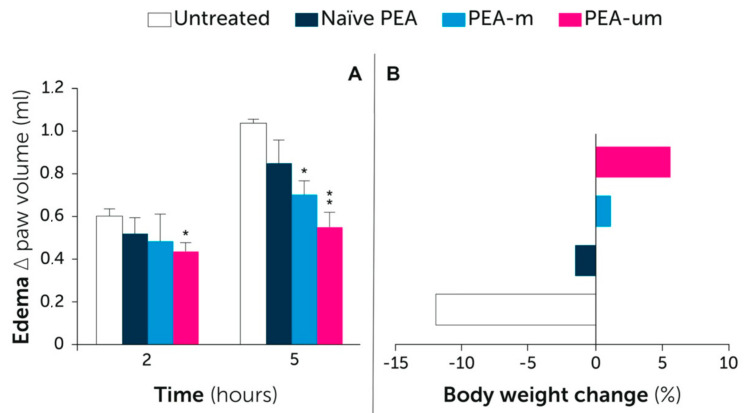
Superior activity of PEA-um compared to other PEA formulations. (A) Effect on inflammatory edema. Only the most relevant time points are depicted. See text for further details. * *p* < 0.05 and ** *p* < 0.01 vs. untreated group. Modified from [124]. (B) Effect on colitis-induced change in body weight. Modified from [125]. Naïve PEA: non-micronized PEA; PEA-m: micronized PEA; PEA-um: ultramicronized PEA.

A major benefit of ultra-micronization is the enhancement of the dissolution rate [122], and better oral absorption [121,122,123]. After oral administration of PEA-um, the plasma concentration of PEA was found to reach a 5-fold higher level than non-micronized (naïve) PEA [120]. Moreover, the oral administration of PEA-um to animals with experimental inflammation resulted in a much higher increase of PEA plasma levels compared to non-inflamed animals, and this was not observed after naïve PEA administration [120]. This suggests that PEA-um provides considerably higher protective power than other forms of PEA under conditions of need. Specifically, in dogs, it was also found that a single dose of PEA-um increased the plasma concentration of PEA by about 5 times, reaching the maximum peak between 1 and 2 h [77]. The higher oral bioavailability obtained following ultra-micronization translated into superior effects, as shown, for example, in a rat model of inflammatory pain [124]. In particular, the study compared micronized, ultra-micronized, and naïve PEA. While no significant differences were observed among treatment groups following intraperitoneal administration, oral supplementation with PEA-um reduced paw edema, local neutrophil infiltration, the histological score of tissue damage, and thermal hyperalgesia to a significantly higher extent compared to micronized and, even more so, naïve PEA [124]. In addition, a study on the enteroprotective benefit of PEA revealed a similar order of magnitude following oral administration, i.e., PEA-um > micronized PEA > naïve PEA [125]. Most notably, a few days after colitis induction, untreated animals experienced diarrhea and a significant reduction in body weight, which was modestly limited by naïve PEA, prevented by micronized PEA, and significantly counteracted by PEA-um [125]. The main findings of the above studies are summarized in Figure 6.

## 8. Application of ALIAmides to Animal Health and Wellbeing

In the last two decades, PEA and related NAEs have been extensively investigated due to the variety of their biological effects [62,67,73,126,127,128,129,130] and lack of toxicity. Micro-PEA of a defined particle size (0.5-10 μm) is safe, the LD50 resulted > 2000 mg/kg body weight for acute oral toxicity and NOEL (no treatment-related adverse effects) greater than 1000 mg/kg body weight for subchronic toxicity [131]. PEA-um has a long track record of use in human and veterinary patients, with good-to-excellent tolerability [62]. Moreover, long-term use of PEA is not associated with the development of tolerance (i.e., a reduction in response after repeated administration) [132,133]. Nowadays, PEA-um is used in several veterinary compositions, like complementary feeds and PARNUTS (feed for particular nutritional purposes) both in the European and North American market. It is sometimes associated with different compounds to better address gastrointestinal, urinary, and skin needs. Moreover, co-micronized mixtures of PEA with natural antioxidants, e.g., quercetin and curcumin, have been developed and are currently used in complementary feeds for dogs and cats in Europe. Finally, topical formulations containing the ALIAmide Adelmidrol, i.e., the diamide derivative of azelaic acid (azeloyl diethanolamide), are also used in pets for skin and mucous membrane health and wellbeing. The main findings obtained so far are presented below.

### 8.1. Gastrointestinal Tract 

Extensive evidence shows that the endocannabinoid system plays a pivotal role in gastrointestinal health [134,135,136,137]. In this respect, PEA has been referred to as an intestinal “gate keeper”, whose increased levels contribute to locking of the gut barrier and to the reduction of intestinal inflammation [135]. Indeed, NAAA inhibition was shown to increase levels of PEA and to reduce inflammation in a mouse model of inflammatory bowel disease (IBD), thereby supporting the “gate keeper” function of PEA [134]. During gut disorders, PEA levels undergo important changes [138,139,140]. In particular, they increase in the colon of dogs with diarrhea [141], while markedly decrease during acute intestinal absorptive disorders and in the course of unbalanced diets [134,142,143]. The enteroprotective effect of PEA passes through the direct or indirect activation of PPAR-α and CB2 receptors [138,139,140,144,145,146,147], whose expression has also been confirmed in the canine and feline gastrointestinal tract [101,109]. It has also recently been found that PEA restores the intestinal barrier permeability via the regulation of intercellular junctions [139,146,148,149,150], and reduces inflammatory cell recruitment, i.e., macrophages and neutrophils, during experimental intestinal injury [138,144,147,151,152,153,154]. PEA administration also decreased viral-induced diarrhea [140] and normalized intestinal motility in a post-inflammatory accelerated transit model [145]. Moreover, a preliminary study in dogs affected with chronic diarrhea demonstrated that dietetic supplementation with PEA reduced the canine IBD activity index (CIBDAI) [141], in line with the findings from Esposito and colleagues (2014), who showed a significant improvement of the colitis activity index (body weight, presence of gross blood in the feces, and stool consistency) in PEA-treated mice with experimentally induced colitis [138]. Finally, a clinical study concluded that dietary supplementation with PEA is able to minimize abdominal pain in human patients affected with irritable bowel syndrome [155]. Almost all of the reported studies have used PEA in the ultra-micronized form, which, as said earlier, is characterized by better bioavailability and a superior enteroprotective effect [125]. A fledgling and very interesting aspect of the endocannabinoid system in gastrointestinal homeostasis is that it interacts with the gut microbiota, with the latter being recently shown to influence the tone of the endocannabinoid system [156]. In turn, deletion in adipose tissue of the gene encoding one of the key enzymes in the NAEs biosynthetic pathways *(Napepld)* profoundly shifts the composition of the gut microbiota, thus providing strong support for the function of PEA and congeners in maintaining microbiota homeostasis [157]. Indeed, PEA administration was found to counteract the specific changes in gut bacterial taxa induced by vitamin D deficiency [158]. A trend towards normalization of the intestinal microbiota profile was also observed by Cristiano and colleagues following PEA treatment in a murine model of autism spectrum disorders [150]. Similarly, the modulation of gut microbiota composition was also observed following sub-chronic treatment with the PEA congener OEA [159]. Intriguingly, a recent paper reported increased levels of NAEs in the stool of IBD patients and mice with experimentally induced colitis [160]. Surprisingly, the authors also found a growth-promoting effect of NAEs on gut bacteria, thus supporting the ability of microbiota, some taxa at least, to catabolize (and purportedly even synthesize) NAEs [160]. Although some preliminary efforts have been made, the role of NAEs and endocannabinoids in host–microbiota homeostasis is far from clear and further studies are needed to address this challenging area and also gain new knowledge on potential synergies between PEA and probiotics.

### 8.2. Upper and Lower Urinary Tract

Great attention has recently been paid to the endocannabinoid system and related NAEs in the regulation of urinary tract homeostasis [161,162,163]. In the kidney, PEA is physiologically present from the embryonic stage [91,164] and is naturally produced in healthy and disease conditions [165,166]. Moreover, PEA levels change in response to kidney damage [167]. The involvement of PEA in the maintenance of renal homeostasis depends on either direct interaction with PPAR-α and GPR55 receptors [168,169,170], or the local increase of the endocannabinoid 2-AG, endowed with nephroprotective function [87,91,171]. Recent studies from our group suggest PEA to be part of a defensive strategy, i.e., monitoring the behavior of renal mast cells, as to keep their mediator release (e.g., chymase) within the physiological range and maintain renal homeostasis accordingly [168]. PEA administration decreases both renal dysfunction and injury triggered by ischemia/reperfusion or contrast agent, besides re-establishing the main markers of glomerular function, i.e., creatinine and urea [168,172]. Moreover, we also found that PEA protects renal blood vessels thanks to its ability to reduce the expression of endothelial adhesion molecules and proinflammatory transcription factors (NF-kB) [168]. These studies highlighted the role of PEA in preserving the physiological features and function of kidneys during adverse conditions [168,172]. Similar results were also reported by Mattace Raso and colleagues (2013) [173], who also showed that PEA reduces blood pressure and kidney damage secondary to hypertension and downregulates angiotensin receptor 1 and angiotensin converting enzyme expression, with a reduction in angiotensin II-mediated effects [173]. Finally, we found that lower doses of PEA are needed to exert a nephroprotective function if natural antioxidant compounds, like silymarin, are concurrently administered [174]. As far as lower urinary tract concerns, PEA levels have been quantified in the bladder and urine [29,175] and found to increase in different settings [175,176]. Moreover, based on the results obtained in experimental models of cystitis, supplementation with PEA is able to restore the normal micturition threshold (i.e., the intra-bladder volume required to stimulate the urinary contraction) [177,178] and reduce visceral pain [177,178,179,180]. Interestingly, a dietetic supplement containing PEA-um was recently described to benefit a Syrian hamster with urolithiasis and diminish recurrence after surgical treatment [181].

### 8.3. Nervous System

Nociception is a complex physiological process encoding and processing noxious stimuli. In particular, noxious stimuli (i.e., unpleasant sensations of mechanical, chemical, and thermal origin) are transformed into electrical signals (transduction), transmitted to the spinal cord (transmission), where they are modulated (modulation) before reaching the brain to be processed (perception/integration) and finally activate the complex and subjective experience of pain [182]. PEA administration was long shown to control nociception [183,184,185]. Most notably, PEA may help set the threshold for nociception by regulating the baseline transcriptional activity of the NF-κB complex [184]. Thanks to this function, PEA was shown to reduce pain in different models of inflammatory and neuropathic pain [62,67,128] as well as human patients (reviewed in [128,186]), the NNT value (numbers needed to treat) for low back pain being 1.7, considerably better than first-line pain-relieving drugs [187]. Pain relief mainly relies on the ability of PEA to downmodulate immune-inflammatory cells, i.e., microglia and mast cells [80,81,188,189,190,191]. The microcomposite resulting from the joint micronization of PEA and the antioxidant polyphenol quercetin (PEA-Q) was recently discovered to alleviate inflammatory pain after a single oral dose [192]. Mixed persistent pain (i.e., involving both inflammatory and neuropathic pain-processing mechanisms) was also found to benefit from PEA-Q dietary supplementation, as shown by a small, albeit interesting, open-field trial on privately owned dogs [193]. 

### 8.4. Musculoskeletal System

N-palmitoyl-D-glucosamine (PGA) is a recently identified component of the ALIAmide family, developed to address orthopedic painful conditions, osteoarthritis in the first place. PGA is a PEA analogue, ethanolamide being substituted with glucosamine, and incorporates both the chondroprotective effects of the latter and the ALIAmide function of the whole molecule. The effect of PGA in a reliable animal model of osteoarthritis pain (i.e., the intraarticular injection of monosodium iodoacetate, MIA) [194,195] has been investigated. In the MIA-injected animals, a single oral administration of PGA resulted in a significant relief of mechanical allodynia (one of the prominent symptoms of neuropathic pain), the effect being enhanced by repeated administration (14 days) and not relying on the glucosamine content [196]. Moreover, oral supplementation with PGA significantly improved the motor functional profile, compared with non-treated animals [196]. In addition, we recently investigated the effect of micronized PGA (PGA-m) on chondrodegeneration, inflammation, and pain in the MIA model of osteoarthritis [197]. Micronized PGA resulted in a superior activity to PGA on MIA-induced mechanical allodynia, locomotor disability, and histologic as well as radiographic damage. The MIA-induced increase in joint mast cells and serum level of proinflammatory and nociceptive mediators was also counteracted by PGA and to a significantly greater extent by m-PGA [197]. Finally, a novel composite developed by co-micronizing PGA and curcumin was found to significantly reduce the severity of cartilage and radiographic damage, as well as osteoarthritis pain in MIA-injected animals [198].

### 8.5. Mucocutaneous and Skin Sites

The diethanolamide of azelaic acid, i.e., Adelmidrol, is a component of the ALIAmide family, possessing both hydrophobic and hydrophilic features, which make it suitable for topical applications. Adelmidrol was found to exert proresolving effects in different models of both acute and chronic inflammation [164,199,200]. When topically applied on canine experimental skin wounds, Adelmidrol downmodulated mast cell degranulation [201], with the effect being paralleled by improved healing [202]. Similar results have recently been obtained by our group in an animal model of diabetic ulcers [203]. An Adelmidrol-containing emulsion was successfully used in pediatric patients with mild atopic dermatitis [204] as well as human patients with giant vulvar syringomas and prominent pruritus [205]. Beagle dogs affected with skin hypersensitivity also benefited from Adelmidrol topical use (i.e., smaller allergic wheals), the compound being locally effective in downmodulating the behavior of skin mast cells [206]. The mechanism is possibly responsible for the decreased severity of pruritus and erythema observed in privately owned dogs with atopic dermatitis, topically treated with Adelmidrol over a 30-day period [207]. A further interesting finding of the study was that owner-evaluated body odor and quality of life also improved [207]. Besides dermatology, dentistry is also one of the fields where Adelmidrol proved to be useful. In client-owned dogs, repeated application of an Adelmidrol mucoadhesive gel, in combination with dental prophylaxis, resulted in less gingival inflammation and a longer duration of dental scaling benefits [208]. Interestingly, it has recently been shown that Adelmidrol increases PEA levels in canine keratinocytes [164]. One might hypothesize that the protective effects presented above depend on the local increase of PEA at sites of Adelmidrol application. PEA, for its part, has repeatedly shown proresolving effects in skin disorders, being able to (i) reduce inflammation in experimental contact allergic dermatitis [79], (ii) downregulate the release of inflammatory chemokine by challenged keratinocytes [79], (iii) decrease transepidermal water loss (TEWL) in human patients [209], and (iv) diminish itching behavior in a mouse model of allergic dermatitis [210]. Interesting data have also been gained in the veterinary side. Dietary supplementation with PEA decreased allergic wheal development in hypersensitive Beagle dogs [77] and delayed the development of clinical signs in dogs with experimental allergic dermatitis [211]. In a multicentric clinical study on 160 privately owned dogs with mild-to-moderate and non-seasonal atopic dermatitis (i.e., the severity usually managed with non-pharmacologic measures, like essential fatty acids), the oral administration of PEA-um over 56 days significantly decreased pruritus and skin lesions, while improving quality of life [212]. Dietary supplementation with micronized PEA proved to benefit cats too, improving skin signs and symptoms in cats with eosinophilic plaque and eosinophilic granuloma [78]. Moreover, a double-blinded placebo-controlled study in allergic cats has recently shown that PEA-um helped to maintain standard therapy-induced remission in cats with hypersensitivity dermatitis, i.e., the so-called proactive approach [213]. Interestingly, during concomitant steroid treatment, the severity of pruritus was significantly lower in the PEA-um-treated cats compared to the placebo, suggesting a possible additional steroid-sparing effect [213].

## 9. Conclusions

**Figure 7:**
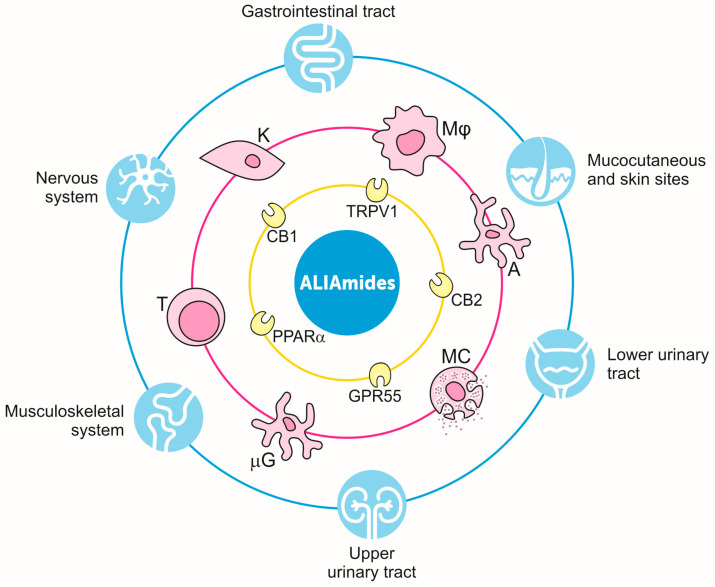
ALIAmides for animal health from a Galileian perspective. Schematic representation of the multitarget function of ALIAmides. Directly and indirectly acting through multiple cannabinoid receptors (yellow ring), PEA and related ALIAmides target different cell populations (pink ring), supporting health maintenance in a variety of body systems (light blue ring). See text for further details. A, astrocyte; CB1, cannabinoid receptor type 1; CB2, cannabinoid receptor type 2; GPR55, G-protein-coupled receptor 55; K, keratinocyte; MC, mast cell; MΦ, macrophage; μG, microglia; PPARα, peroxisome proliferator-activated receptor α; T, T cells; TRPV1, transient receptor potential vanilloid 1.

PEA and related amides belong to a class of physiological compounds, i.e., ALIAmides, commonly present in plants and animal food sources. Most ALIAmides are body-own components, locally produced “on demand” for homeostatic purposes. A great deal of evidence supports the proresolving function of endogenous PEA and related lipid amides. The multitarget actions of these natural bioacting compounds, their mutual interactions, and the close relationship with the endocannabinoid system are the key features for meeting the health needs of different body systems (Figure 7). The health-promoting effects resulting from the use of ALIAmides, provided they are administered in a bioavailable form, together with the safety and tolerability profile, led to the development of complementary feeds, PARNUTS, and topical products for the maintenance of heath in companion animals. The positive results obtained so far may encourage stronger efforts to further investigate the potential benefits arising from ALIAmides as a response to different animals’ needs, health, and wellbeing.

## Figures and Tables

**Table 1:** Food sources in order of decreasing concentration of palmitoyl ethanolamide (PEA).

Food Source	ng/g f.w.	Reference
Soy lecithin	950,000	[23]
Soybean (*Glycine max*)	6700	[23,24]
Green coffee (depending on the variety)	2830-11,940	[27]
Raw peanuts	~7770	[26]
Roasted coffee	7200	[27]
Peanuts (*Arachis hypogaea*)	3730	[23,24]
Alfalfa (*Medicago sativa*)	1150	[24]
Refined wheat flour	~ 800 *	[25]
Whole wheat flour	~ 400 *	[25]
Raw pearl barley	~330	[26]
Walnuts	~ 250 *	[25]
Toasted pearl barley	~220	[26]
Corn	200	[23]
Black-eyed peas (*Vigna unguiculata*)	138	[24]
Broccoli	~130 *	[25]
Tuna fish	~120 *	[25]
Chicken	~120 *	[25]
Carrots	~110 *	[25]
Eggs	~ 100 *	[25]
Tomato	100	[23]
Garden pea (*Pisum sativum*)	100	[23,24]
Beans	~90 *	[25]
Lettuce	~70 *	[25]
Beef	~ 60 *	[25]
Codfish	~ 60 *	[25]
Common bean (*Phaseolus vulgaris*)	53.5	[24]
Cauliflowers	~50 *	[25]
Chickpeas	~40 *	[25]
Anchovies	~ 40 *	[25]
Cow’s milk	~ 30 *	[25]
Almonds	~ 10 *	[25]
Grapes	~10 *	[25]
Oranges	~10 *	[25]
Apples	~8 *	[25]
Lentils	~6 *	[25]
Potatoes	5 *	[25]
Elk milk	1.81	[30]
Bovine milk	0.25	[30]

f.w. = fresh-weight; * = the concentration is expressed as nanograms per gram on a dry-weight basis; ~ indicates the value has been extrapolated from figures.

**Table 2:** A glance over cannabinoid receptor distribution in dogs and cats [95,96,97,98,99,100,101,102,103,104,105,106,107,108,109].

SYSTEM/TISSUE	AREA/CELL	CB1	CB2	GPR55	PPARα	TRPV1
CNS	Hippocampus	⬤				
	Claustrum	◓				
	Cerebral cortex	◓				
	Cornu Ammonis	◓				
	Midbrain	◓				
	Cerebellum	◓				
	Medulla oblongata	◓				
	Spinal cord	◓				
	Spinal glial cells		◓			
	Astrocytes	◓	◓			
	Cerebral arterial smooth muscle cells	◒				
PNS	Dorsal root ganglia (neurons, satellite cells)	◓				
	Schwann cells	◓				
SKIN	Dermal papillae				◒	
	Hair follicles	◓	◓		◒	
	Hair bulb cells	◒	◒			
	Sebaceous glands	⬤	⬤			
	Sweat glands	◓	⬤			
	Keratinocytes	⬤	⬤		◒	◓
	Mast cells	◓	◓			
	Fibroblasts	◓	◓			
GI TRACT	Salivary glands	◓				
	Lamina propria cells	◓				
	Enterocytes	◓	◒			
	Mast cells		◓			
	Immunocytes	◓	◓	◒	⬤	
	Smooth muscle cells		◓	⬤	⬤	
	Macrophages		◒	⬤		
	Submucosal plexus (neurons and glial cells)		◓			
	Myenteric plexus glial cells				◓	
	Myenteric plexus neurons			◒		
	Intestinal enteroendocrine cells	◒	◒	◒		
	Goblet cells	◒				
	Enteric neurons	◒				
	Enteroglial cells				◒	
CIRCULATORY SYSTEM	Lymph nodes		⬤			
	B cells		◓			
	Endothelial cells		◓		◓	
	Spleen		◓			
GENITAL TRACT	Ovary	◒				
	Oviducts	◒				

◒ Cat; ◓ Dog; ⬤ Cat and dog; CIRCULATORY SYSTEM = blood vascular and lymphatic system; CNS = central nervous system; PNS = peripheral nervous system; GI TRACT = gastrointestinal tract; CB1= Cannabinoid receptor type 1; CB2= Cannabinoid receptor type 2; GPR55 = G protein-coupled receptor 55; PPAR-α = Peroxisome proliferator-activated receptor alpha; TRPV1= Transient receptor potential vanilloid 1.

## References

[1] Zohoori F.V. (2020). Nutrition and Diet Monogr. Oral. Sci..

[2] Panagiotou G., Nielsen J. (2009). Nutritional systems biology: Definitions and approaches. Annu. Rev. Nutr..

[3] Davies M. (2016). Veterinary clinical nutrition: Success stories: An overview. Proc. Nutr. Soc..

[4] Gupta R.C., Srivastava A., Lall R. (2019). Nutraceuticals in Veterinary Medicine.

[5] Saevik B.K., Bergvall K., Holm B.R., Saijonmaa-Koulumies L.E., Hedhammar A., Larsen S., Kristensen F. (2004). A randomized, controlled study to evaluate the steroid sparing effect of essential fatty acid supplementation in the treatment of canine atopic dermatitis. Vet. Dermatol..

[6] Schumann J., Basiouni S., Gück T., Fuhrmann H. (2014). Treating canine atopic dermatitis with unsaturated fatty acids: The role of mast cells and potential mechanisms of action. J. Anim. Physiol. Anim. Nutr. (Berl).

[7] Witzel-Rollins A., Murphy M., Becvarova I., Were S.R., Cadiergues M.C., Meyer H. (2019). Non-controlled, open-label clinical trial to assess the effectiveness of a dietetic food on pruritus and dermatologic scoring in atopic dogs. BMC Vet. Res..

[8] Plantinga E.A., Everts H., Kastelein A.M., Beynen A.C. (2005). Retrospective study of the survival of cats with acquired chronic renal insufficiency offered different commercial diets. Vet. Rec..

[9] Brown S.A., Brown C.A., Crowell W.A., Barsanti J.A., Allen T., Cowell C., Finco D.R. (1998). Beneficial effects of chronic administration of dietary omega-3 polyunsaturated fatty acids in dogs with renal insufficiency. J. Lab. Clin. Med..

[10] Pan Y., Landsberg G., Mougeot I., Kelly S., Xu H., Bhatnagar S., Gardner C.L., Milgram N.W. (2018). Efficacy of a therapeutic diet on dogs with signs of cognitive dysfunction syndrome (CDS): A prospective double blinded placebo controlled clinical study. Front. Nutr..

[11] Mehler S.J., May L.R., King C., Harris W.S., Shah Z. (2016). A prospective, randomized, double blind, placebo-controlled evaluation of the effects of eicosapentaenoic acid and docosahexaenoic acid on the clinical signs and erythrocyte membrane polyunsaturated fatty acid concentrations in dogs with osteoarthritis. Prostaglandins Leukot. Essent. Fatty Acids..

[12] Fritsch D.A., Allen T.A., Dodd C.E., Jewell D.E., Sixby K.A., Leventhal P.S., Brejda J., Hahn K.A. (2010). A multicenter study of the effect of dietary supplementation with fish oil omega-3 fatty acids on carprofen dosage in dogs with osteoarthritis. J. Am. Vet. Med. Assoc..

[13] Moreau M., Troncy E., Del Castillo J.R., Bédard C., Gauvin D., Lussier B. (2013). Effects of feeding a high omega-3 fatty acids diet in dogs with naturally occurring osteoarthritis. J. Anim. Physiol. Anim. Nutr..

[14] Rialland P., Bichot S., Lussier B., Moreau M., Beaudry F., del Castillo J.R., Gauvin D., Troncy E. (2013). Effect of a diet enriched with green-lipped mussel on pain behavior and functioning in dogs with clinical osteoarthritis. Can. J. Vet. Res..

[15] Roush J.K., Dodd C.E., Fritsch D.A., Allen T.A., Jewell D.E., Schoenherr W.D., Richardson D.C., Leventhal P.S., Hahn K.A. (2010). Multicenter veterinary practice assessment of the effects of omega-3 fatty acids on osteoarthritis in dogs. J. Am. Vet. Med. Assoc..

[16] Alves J.C., Santos A.M., Jorge P.I. (2017). Effect of an oral joint supplement when compared to carprofen in the management of hip osteoarthritis in working dogs. Top. Companion. Anim. Med..

[17] Bhathal A., Spryszak M., Louizos C., Frankel G. (2017). Glucosamine and chondroitin use in canines for osteoarthritis: A review. Open Vet. J..

[18] McCarthy G., O’donovan J., Jones B., McAllister H., Seed M., Mooney C. (2007). Randomised double-blind, positive-controlled trial to assess the efficacy of glucosamine/chondroitin sulfate for the treatment of dogs with osteoarthritis. Vet. J..

[19] Musco N., Vassalotti G., Mastellone V., Cortese L., Della Rocca G., Molinari M.L., Calabrò S., Tudisco R., Cutrignelli M.I., Lombardi P. (2019). Effects of a nutritional supplement in dogs affected by osteoarthritis. Vet. Med. Sci..

[20] Coburn A.F., Trulson M.F., Moore L.V. (1954). [Further study of the effect of the administration of egg yolk on susceptibility of children to rheumatic infection]. Minerva Med..

[21] Ganley O.H., Graessle O.E., Robinson H.J. (1958). Anti-inflammatory activity on compounds obtained from egg yolk, peanut oil, and soybean lecithin. J. Lab. Clin. Med..

[22] Kuehl F.A., Jacob T.A., Ganley O.H., Ormond R.E., Meisinger M.A.P. (1957). The identification of N-(2-hydroxyethyl)-palmitamide as a naturally occuring anti-inflammatory agent. J. Am. Chem. Soc..

[23] Kilaru A., Blancaflor E.B., Venables B.J., Tripathy S., Mysore K.S., Chapman K.D. (2007). The N-acylethanolamine-mediated regulatory pathway in plants. Chem. Biodivers..

[24] Venables B.J., Waggoner C.A., Chapman K.D. (2005). N-acylethanolamines in seeds of selected legumes. Phytochemistry.

[25] De Luca L., Ferracane R., Vitaglione P. (2019). Food database of N-acyl-phosphatidylethanolamines, N-acylethanolamines and endocannabinoids and daily intake from a Western, a Mediterranean and a vegetarian diet. Food Chem..

[26] Cordaro M., Scuto M., Siracusa R., D’amico R., Peritore F.A., Gugliandolo E., Fusco R., Crupi R., Impellizzeri D., Pozzebon M. (2020). Effect of N-palmitoylethanolamine-oxazoline on comorbid neuropsychiatric disturbance associated with inflammatory bowel disease. FASEB J..

[27] Impellizzeri D., Cordaro M., Bruschetta G., Crupi R., Pascali J., Alfonsi D., Marcolongo G., Cuzzocrea S. (2016). 2-pentadecyl-2-oxazoline: Identification in coffee, synthesis and activity in a rat model of carrageenan-induced hindpaw inflammation. Pharmacol. Res..

[28] Schuel H., Burkman L.J., Lippes J., Crickard K., Forester E., Piomelli D., Giuffrida A. (2002). N-Acylethanolamines in human reproductive fluids. Chem. Phys. Lipids.

[29] Lam P.M., Marczylo T.H., Konje J.C. (2010). Simultaneous measurement of three N-acylethanolamides in human bio-matrices using ultra performance liquid chromatography-tandem mass spectrometry. Anal. Bioanal. Chem..

[30] Gouveia-Figueira S., Nording M.L. (2014). Development and validation of a sensitive UPLC-ESI-MS/MS method for the simultaneous quantification of 15 endocannabinoids and related compounds in milk and other biofluids. Anal. Chem..

[31] Gaitán A.V., Wood J.T., Solomons N.W., Donohue J.A., Ji L., Liu Y., Nikas S.P., Zhang F., Allen L.H., Makriyannis A. (2019). Endocannabinoid metabolome characterization of milk from guatemalan women living in the western highlands. Curr. Dev. Nutr..

[32] Bruun S., Gouveia-Figueira S., Domellöf M., Husby S., Neergaard Jacobsen L., Michaelsen K.F., Fowler C.J., Zachariassen G. (2018). Satiety factors oleoylethanolamide, stearoylethanolamide, and palmitoylethanolamide in mother’s milk are strongly associated with infant weight at four months of age-data from the odense child cohort. Nutrients.

[33] Ramírez-López M.T., Vázquez M., Lomazzo E., Hofmann C., Blanco R.N., Alén F., Antón M., Decara J., Arco R., Orio L. (2017). A moderate diet restriction during pregnancy alters the levels of endocannabinoids and endocannabinoid-related lipids in the hypothalamus, hippocampus and olfactory bulb of rat offspring in a sex-specific manner. PLoS ONE.

[34] Bachur N.R., Masek K., Melmon K.L., Udenfriend S. (1965). Fatty acid amides of ethanolamine in mammalian tissues. J. Biol. Chem..

[35] Epps D.E., Schmid P.C., Natarajan V., Schmid H.H. (1979). N-Acylethanolamine accumulation in infarcted myocardium. Biochem. Biophys. Res. Commun..

[36] Abramo F., Campora L., Albanese F., della Valle M.F., Cristino L., Petrosino S., Di Marzo V., Miragliotta V. (2014). Increased levels of palmitoylethanolamide and other bioactive lipid mediators and enhanced local mast cell proliferation in canine atopic dermatitis. BMC Vet. Res..

[37] Matias I., Wang J.W., Moriello A.S., Nieves A., Woodward D.F., Di Marzo V. (2006). Changes in endocannabinoid and palmitoylethanolamide levels in eye tissues of patients with diabetic retinopathy and age-related macular degeneration. Prostaglandins Leukot. Essent. Fatty Acids..

[38] Annuzzi G., Piscitelli F., Di Marino L., Patti L., Giacco R., Costabile G., Bozzetto L., Riccardi G., Verde R., Petrosino S. (2010). Differential alterations of the concentrations of endocannabinoids and related lipids in the subcutaneous adipose tissue of obese diabetic patients. Lipids Health Dis..

[39] Schreiber D., Harlfinger S., Nolden B.M., Gerth C.W., Jaehde U., Schomig E., Klosterkotter J., Giuffrida A., Astarita G., Piomelli D. (2007). Determination of anandamide and other fatty acyl ethanolamides in human serum by electrospray tandem mass spectrometry. Anal. Biochem..

[40] Baker D., Pryce G., Croxford J.L., Brown P., Pertwee R.G., Makriyannis A., Khanolkar A., Layward L., Fezza F., Bisogno T. (2001). Endocannabinoids control spasticity in a multiple sclerosis model. FASEB J..

[41] Artmann A., Petersen G., Hellgren L.I., Boberg J., Skonberg C., Nellemann C., Hansen S.H., Hansen H.S. (2008). Influence of dietary fatty acids on endocannabinoid and N-acylethanolamine levels in rat brain, liver and small intestine. Biochim. Biophys. Acta.

[42] Bisogno T., Martire A., Petrosino S., Popoli P., Di Marzo V. (2008). Symptom-related changes of endocannabinoid and palmitoylethanolamide levels in brain areas of R6/2 mice, a transgenic model of Huntington’s disease. Neurochem. Int..

[43] Kilaru A., Isaac G., Tamura P., Baxter D., Duncan S.R., Venables B.J., Welti R., Koulen P., Chapman K.D. (2010). Lipid profiling reveals tissue-specific differences for ethanolamide lipids in mice lacking fatty acid amide hydrolase. Lipids.

[44] Franklin A., Parmentier-Batteur S., Walter L., Greenberg D.A., Stella N. (2003). Palmitoylethanolamide increases after focal cerebral ischemia and potentiates microglial cell motility. J. Neurosci..

[45] Muccioli G.G., Stella N. (2008). An optimized GC-MS method detects nanomolar amounts of anandamide in mouse brain. Anal. Biochem..

[46] Schmid P.C., Krebsbach R.J., Perry S.R., Dettmer T.M., Maasson J.L., Schmid H.H. (1995). Occurrence and postmortem generation of anandamide and other long-chain N-acylethanolamines in mammalian brain. FEBS Lett..

[47] Richardson D., Ortori C.A., Chapman V., Kendall D.A., Barrett D.A. (2007). Quantitative profiling of endocannabinoids and related compounds in rat brain using liquid chromatography-tandem electrospray ionization mass spectrometry. Anal. Biochem..

[48] Rubio M., McHugh D., Fernández-Ruiz J., Bradshaw H., Walker J.M. (2007). Short-term exposure to alcohol in rats affects brain levels of anandamide, other N-acylethanolamines and 2-arachidonoyl-glycerol. Neurosci. Lett..

[49] Fonseca B.M., Correia-da-Silva G., Taylor A.H., Lam P.M., Marczylo T.H., Konje J.C., Bell S.C., Teixeira N.A. (2010). N-acylethanolamine levels and expression of their metabolizing enzymes during pregnancy. Endocrinology.

[50] Richardson D., Pearson R.G., Kurian N., Latif M.L., Garle M.J., Barrett D.A., Kendall D.A., Scammell B.E., Reeve A.J., Chapman V. (2008). Characterisation of the cannabinoid receptor system in synovial tissue and fluid in patients with osteoarthritis and rheumatoid arthritis. Arthritis Res. Ther..

[51] Valastro C., Campanile D., Marinaro M., Franchini D., Piscitelli F., Verde R., Di Marzo V., Di Bello A. (2017). Characterization of endocannabinoids and related acylethanolamides in the synovial fluid of dogs with osteoarthritis: A pilot study. BMC Vet. Res..

[52] Aloe L., Leon A., Levi-Montalcini R. (1993). A proposed autacoid mechanism controlling mastocyte behaviour. Agents Actions.

[53] Melmon K.L., Rocklin R.E., Rosenkranz R.P. (1981). Autacoids as modulators of the inflammatory and immune response. Am. J. Med..

[54] Chiurchiù V., Leuti A., Maccarrone M. (2018). Bioactive lipids and chronic inflammation: Managing the fire within. Front. Immunol..

[55] Chapman K.D. (2000). Emerging physiological roles for N-acylphosphatidylethanolamine metabolism in plants: Signal transduction and membrane protection. Chem. Phys. Lipids.

[56] Skaper S.D., Facci L., Giusti P. (2014). Mast cells, glia and neuroinflammation: Partners in crime?. Immunology.

[57] Harrison N., Lone M.A., Kaul T.K., Reis Rodrigues P., Ogungbe I.V., Gill M.S. (2014). Characterization of N-acyl phosphatidylethanolamine-specific phospholipase-D isoforms in the nematode Caenorhabditis elegans. PLoS ONE.

[58] Schmid H.H., Berdyshev E.V. (2002). Cannabinoid receptor-inactive N-acylethanolamines and other fatty acid amides: Metabolism and function. Prostaglandins Leukot. Essent. Fatty Acids..

[59] Chapman K.D. (2004). Occurrence, metabolism, and prospective functions of N-acylethanolamines in plants. Prog. Lipid Res..

[60] Muccioli G.G., Sia A., Muchowski P.J., Stella N. (2009). Genetic manipulation of palmitoylethanolamide production and inactivation in Saccharomyces cerevisiae. PLoS ONE.

[61] Sepe N., De Petrocellis L., Montanaro F., Cimino G., Di Marzo V. (1998). Bioactive long chain N-acylethanolamines in five species of edible bivalve molluscs. Possible implications for mollusc physiology and sea food industry. Biochim. Biophys. Acta.

[62] Petrosino S., Di Marzo V. (2017). The pharmacology of palmitoylethanolamide and first data on the therapeutic efficacy of some of its new formulations. Br. J. Pharmacol..

[63] Hussain Z., Uyama T., Tsuboi K., Ueda N. (2017). Mammalian enzymes responsible for the biosynthesis of N-acylethanolamines. Biochim. Biophys. Acta Mol. Cell Biol. Lipids.

[64] Bisogno T. (2008). Endogenous cannabinoids: Structure and metabolism. J. Neuroendocrinol..

[65] Tsuboi K., Ikematsu N., Uyama T., Deutsch D.G., Tokumura A., Ueda N. (2013). Biosynthetic pathways of bioactive N-acylethanolamines in brain. CNS Neurol. Disord. Drug Targets.

[66] Balvers M.G., Verhoeckx K.C., Meijerink J., Wortelboer H.M., Witkamp R.F. (2013). Measurement of palmitoylethanolamide and other N-acylethanolamines during physiological and pathological conditions. CNS Neurol. Disord. Drug Targets.

[67] Esposito E., Cuzzocrea S. (2013). Palmitoylethanolamide in homeostatic and traumatic central nervous system injuries. CNS Neurol. Disord. Drug Targets.

[68] Hansen H.S. (2013). Effect of diet on tissue levels of palmitoylethanolamide. CNS Neurol. Disord. Drug Targets.

[69] Berdyshev E.V., Schmid P.C., Dong Z., Schmid H.H. (2000). Stress-induced generation of N-acylethanolamines in mouse epidermal JB6 P+ cells. Biochem. J..

[70] Alhouayek M., Muccioli G.G. (2014). Harnessing the anti-inflammatory potential of palmitoylethanolamide. Drug Discov. Today..

[71] Rinne P., Guillamat-Prats R., Rami M., Bindila L., Ring L., Lyytikäinen L.P., Raitoharju E., Oksala N., Lehtimäki T., Weber C. (2018). Palmitoylethanolamide promotes a proresolving macrophage phenotype and attenuates atherosclerotic plaque formation. Arterioscler. Thromb. Vasc. Biol..

[72] Roviezzo F., Rossi A., Caiazzo E., Orlando P., Riemma M.A., Iacono V.M., Guarino A., Ialenti A., Cicala C., Peritore A. (2017). Palmitoylethanolamide supplementation during sensitization prevents airway allergic symptoms in the mouse. Front. Pharmacol..

[73] Skaper S.D., Facci L., Barbierato M., Zusso M., Bruschetta G., Impellizzeri D., Cuzzocrea S., Giusti P. (2015). N-Palmitoylethanolamine and neuroinflammation: A novel therapeutic strategy of resolution. Mol. Neurobiol..

[74] Solorzano C., Zhu C., Battista N., Astarita G., Lodola A., Rivara S., Mor M., Russo R., Maccarrone M., Antonietti F. (2009). Selective N-acylethanolamine-hydrolyzing acid amidase inhibition reveals a key role for endogenous palmitoylethanolamide in inflammation. Proc. Natl. Acad. Sci. USA.

[75] Cerrato S., Brazis P., Miolo A., della Valle M.F., Puigdemont A. (2010). Effects of palmitoylethanolamide on immunologically induced histamine, PGD2 and TNFα release from canine skin mast cells. Vet. Immunol. Immunopathol..

[76] Abramo F., Lazzarini G., Pirone A., Lenzi C., Albertini S., della Valle M.F., Schievano C., Vannozzi I., Miragliotta V. (2017). Ultramicronized palmitoylethanolamide counteracts the effects of compound 48/80 in a canine skin organ culture model. Vet. Dermatol..

[77] Cerrato S., Brazis P., della Valle M.F., Miolo A., Petrosino S., Di Marzo V., Puigdemont A. (2012). Effects of palmitoylethanolamide on the cutaneous allergic inflammatory response in Ascaris hypersensitive Beagle dogs. Vet. J..

[78] Scarampella F., Abramo F., Noli C. (2001). Clinical and histological evaluation of an analogue of palmitoylethanolamide, PLR 120 (comicronized Palmidrol INN) in cats with eosinophilic granuloma and eosinophilic plaque: A pilot study. Vet. Dermatol..

[79] Petrosino S., Cristino L., Karsak M., Gaffal E., Ueda N., Tüting T., Bisogno T., De Filippis D., D’Amico A., Saturnino C. (2010). Protective role of palmitoylethanolamide in contact allergic dermatitis. Allergy.

[80] Bettoni I., Comelli F., Colombo A., Bonfanti P., Costa B. (2013). Non-neuronal cell modulation relieves neuropathic pain: Efficacy of the endogenous lipid palmitoylethanolamide. CNS Neurol. Disord. Drug Targets.

[81] Luongo L., Guida F., Boccella S., Bellini G., Gatta L., Rossi F., de Novellis V., Maione S. (2013). Palmitoylethanolamide reduces formalin-induced neuropathic-like behaviour through spinal glial/microglial phenotypical changes in mice. CNS Neurol. Disord. Drug Targets.

[82] Guida F., Luongo L., Marmo F., Romano R., Iannotta M., Napolitano F., Belardo C., Marabese I., D’Aniello A., De Gregorio D. (2015). Palmitoylethanolamide reduces pain-related behaviors and restores glutamatergic synapses homeostasis in the medial prefrontal cortex of neuropathic mice. Mol. Brain.

[83] Gabrielsson L., Gouveia-Figueira S., Häggström J., Alhouayek M., Fowler C.J. (2017). The anti-inflammatory compound palmitoylethanolamide inhibits prostaglandin and hydroxyeicosatetraenoic acid production by a macrophage cell line. Pharmacol. Res. Perspect..

[84] Chiurchiù V., Leuti A., Smoum R., Mechoulam R., Maccarrone M. (2018). Bioactive lipids ALIAmides differentially modulate inflammatory responses of distinct subsets of primary human T lymphocytes. FASEB J..

[85] Lo Verme J., Fu J., Astarita G., La Rana G., Russo R., Calignano A., Piomelli D. (2005). The nuclear receptor peroxisome proliferator-activated receptor-alpha mediates the anti-inflammatory actions of palmitoylethanolamide. Mol. Pharmacol..

[86] Ryberg E., Larsson N., Sjögren S., Hjorth S., Hermansson N.O., Leonova J., Elebring T., Nilsson K., Drmota T., Greasley P.J. (2007). The orphan receptor GPR55 is a novel cannabinoid receptor. Br. J. Pharmacol..

[87] Petrosino S., Schiano Moriello A., Cerrato S., Fusco M., Puigdemont A., De Petrocellis L., Di Marzo V. (2016). The anti-inflammatory mediator palmitoylethanolamide enhances the levels of 2-arachidonoyl-glycerol and potentiates its actions at TRPV1 cation channels. Br. J. Pharmacol..

[88] Petrosino S., Schiano Moriello A., Verde R., Allarà M., Imperatore R., Ligresti A., Mahmoud A.M., Peritore A.F., Iannotti F.A., Di Marzo V. (2019). Palmitoylethanolamide counteracts substance P-induced mast cell activation in vitro by stimulating diacylglycerol lipase activity. J. Neuroinflamm..

[89] Di Marzo V., Melck D., Orlando P., Bisogno T., Zagoory O., Bifulco M., Vogel Z., De Petrocellis L. (2001). Palmitoylethanolamide inhibits the expression of fatty acid amide hydrolase and enhances the anti-proliferative effect of anandamide in human breast cancer cells. Biochem. J..

[90] Ho W.S., Barrett D.A., Randall M.D. (2008). Entourage effects of N-palmitoylethanolamide and N-oleoylethanolamide on vasorelaxation to anandamide occur through TRPV1 receptors. Br. J. Pharmacol..

[91] De Petrocellis L., Davis J.B., Di Marzo V. (2001). Palmitoylethanolamide enhances anandamide stimulation of human vanilloid VR1 receptors. FEBS Lett..

[92] Ambrosino P., Soldovieri M.V., De Maria M., Russo C., Taglialatela M. (2014). Functional and biochemical interaction between PPARalpha receptors and TRPV1 channels: Potential role in PPARalpha agonists-mediated analgesia. Pharmacol. Res..

[93] Ambrosino P., Soldovieri M.V., Russo C., Taglialatela M. (2013). Activation and desensitization of TRPV1 channels in sensory neurons by the PPARalpha agonist palmitoylethanolamide. Br. J. Pharmacol..

[94] Silver R.J. (2019). The endocannabinoid system of animals. Animals (Basel).

[95] Barbero R., Vercelli C., Cuniberti B., Martano M., della Valle M.F., Re G. (2018). Expression of functional TRPV1 receptors in primary culture of canine keratinocytes. J. Vet. Pharmacol. Ther..

[96] Campora L., Miragliotta V., Ricci E., Cristino L., Di Marzo V., Albanese F., della Valle M.F., Abramo F. (2012). Cannabinoid receptor type 1 and 2 expression in the skin of healthy dogs and dogs with atopic dermatitis. Am. J. Vet. Res..

[97] Dall’Aglio C., Mercati F., Pascucci L., Boiti C., Pedini V., Ceccarelli P. (2010). Immunohistochemical localization of CB1 receptor in canine salivary glands. Vet. Res. Commun..

[98] Fernández-Trapero M., Espejo-Porras F., Rodríguez-Cueto C., Coates J.R., Pérez-Díaz C., de Lago E., Fernández-Ruiz J. (2017). Upregulation of CB2 receptors in reactive astrocytes in canine degenerative myelopathy, a disease model of amyotrophic lateral sclerosis. Dis. Model. Mech..

[99] Freundt-Revilla J., Kegler K., Baumgärtner W., Tipold A. (2017). Spatial distribution of cannabinoid receptor type 1 (CB1) in normal canine central and peripheral nervous system. PLoS ONE.

[100] Freundt-Revilla J., Heinrich F., Zoerner A., Gesell F., Beyerbach M., Shamir M., Oevermann A., Baumgärtner W., Tipold A. (2018). The endocannabinoid system in canine steroid-responsive meningitis-arteritis and intraspinal spirocercosis. PLoS ONE.

[101] Galiazzo G., Giancola F., Stanzani A., Fracassi F., Bernardini C., Forni M., Pietra M., Chiocchetti R. (2018). Localization of cannabinoid receptors CB1, CB2, GPR55 and PPARalfa in the canine gastrointestinal tract. Histochem. Cell Biol..

[102] Gebremedhin D., Lange A.R., Campbell W.B., Hillard C.J., Harder D.R. (1999). Cannabinoid CB1 receptor of cat cerebral arterial muscle functions to inhibit L-type Ca2+ channel current. Am. J. Physiol..

[103] Mercati F., Dall’Aglio C., Pascucci L., Boiti C., Ceccarelli P. (2012). Identification of cannabinoid type 1 receptor in dog hair follicles. Acta Histochem..

[104] Miragliotta V., Ricci P.L., Albanese F., Pirone A., Tognotti D., Abramo F. (2018). Cannabinoid receptor types 1 and 2 and peroxisome proliferator-activated receptor-alpha: Distribution in the skin of clinically healthy cats and cats with hypersensitivity dermatitis. Vet. Dermatol..

[105] Ndong C., O’Donnell D., Ahmad S., Groblewski T. (2011). Cloning and pharmacological characterization of the dog cannabinoid CB2receptor. Eur. J. Pharmacol..

[106] Pirone A., Cantile C., Miragliotta V., Lenzi C., Giannessi E., Cozzi B. (2016). Immunohistochemical distribution of the cannabinoid receptor 1 and fatty acid amide hydrolase in the dog claustrum. J. Chem. Neuroanat..

[107] Pirone A., Lenzi C., Briganti A., Abbate F., Levanti M., Abramo F., Miragliotta V. (2017). Spatial distribution of cannabinoid receptor 1 and fatty acid amide hydrolase in the cat ovary and oviduct. Acta Histochem..

[108] Ponti W., Rubino T., Bardotti M., Poli G., Parolaro D. (2001). Cannabinoids inhibit nitric oxide production in bone marrow derived feline macrophages. Vet. Immunol. Immunopathol..

[109] Stanzani A., Galiazzo G., Giancola F., Tagliavia C., De Silva M., Pietra M., Fracassi F., Chiocchetti R. (2020). Localization of cannabinoid and cannabinoid related receptors in the cat gastrointestinal tract. Histochem. Cell Biol..

[110] Medzhitov R. (2008). Origin and physiological roles of inflammation. Nature.

[111] Feehan K.T., Gilroy D.W. (2019). Is resolution the end of inflammation?. Trends Mol. Med..

[112] Nathan C., Ding A. (2010). Nonresolving inflammation. Cell.

[113] Serhan C.N. (2014). Pro-resolving lipid mediators are leads for resolution physiology. Nature.

[114] Dinarello C.A., Simon A., van der Meer J.W. (2012). Treating inflammation by blocking interleukin-1 in a broad spectrum of diseases. Nat. Rev. Drug Discov..

[115] Flower R.J. (2006). Prostaglandins, bioassay and inflammation. Br. J. Pharmacol..

[116] Tabas I., Glass C.K. (2013). Anti-inflammatory therapy in chronic disease: Challenges and opportunities. Science.

[117] Cordaro M., Cuzzocrea S., Crupi R. (2020). An update of palmitoylethanolamide and luteolin effects in preclinical and clinical studies of neuroinflammatory events. Antioxidants (Basel).

[118] Lerner R., Pascual Cuadrado D., Post J.M., Lutz B., Bindila L. (2019). Broad lipidomic and transcriptional changes of prophylactic PEA administration in adult mice. Front. Neurosci..

[119] Bilia A.R., Piazzini V., Guccione C., Risaliti L., Asprea M., Capecchi G., Bergonzi M.C. (2017). Improving on nature: The role of nanomedicine in the development of clinical natural drugs. Planta Med..

[120] Petrosino S., Cordaro M., Verde R., Schiano Moriello A., Marcolongo G., Schievano C., Siracusa R., Piscitelli F., Peritore A.F., Crupi R. (2018). Oral ultramicronized palmitoylethanolamide: Plasma and tissue levels and spinal anti-hyperalgesic effect. Front Pharmacol..

[121] Takano R., Furumoto K., Shiraki K., Takata N., Hayashi Y., Aso Y., Yamashita S. (2008). Rate-limiting steps of oral absorption for poorly water-soluble drugs in dogs; prediction from a miniscale dissolution test and a physiologically-based computer simulation. Pharm. Res..

[122] Leleux J., Williams R.O. (2014). Recent advancements in mechanical reduction methods: Particulate systems. Drug. Dev. Ind. Pharm..

[123] Rao S., Song Y., Peddie F., Evans A.M. (2011). Particle size reduction to the nanometer range: A promising approach to improve buccal absorption of poorly water-soluble drugs. Int. J. Nanomed..

[124] Impellizzeri D., Bruschetta G., Cordaro M., Crupi R., Siracusa R., Esposito E., Cuzzocrea S. (2014). Micronized/ultramicronized palmitoylethanolamide displays superior oral efficacy compared to nonmicronized palmitoylethanolamide in a rat model of inflammatory pain. J. Neuroinflamm..

[125] Impellizzeri D., Bruschetta G., Cordaro M., Crupi R., Siracusa R., Esposito E., Cuzzocrea S. (2015). Ultramicronized palmitoylethanolamide reduces inflammation in a Th1-mediated model of colitis. Eur. J. Inflamm..

[126] Artukoglu B.B., Beyer C., Zuloff-Shani A., Brener E., Bloch M.H. (2017). Efficacy of palmitoylethanolamide for pain: A meta-analysis. Pain Phys..

[127] Re G., Barbero R., Miolo A., Di Marzo V. (2007). Palmitoylethanolamide, endocannabinoids and related cannabimimetic compounds in protection against tissue inflammation and pain: Potential use in companion animals. Vet. J..

[128] Skaper S.D., Facci L., Fusco M., della Valle M.F., Zusso M., Costa B., Giusti P. (2014). Palmitoylethanolamide, a naturally-occurring disease modifying agent in neuropathic pain. Inflammopharmacology.

[129] Tsuboi K., Uyama T., Okamoto Y., Ueda N. (2018). Endocannabinoids and related N-acylethanolamines: Biological activities and metabolism. Inflamm. Regen..

[130] Palazzo E., Luongo L., Guida F., de Novellis V., Boccella S., Marabese I., Maione S., Cristiano C. (2018). Role of N-Acylethanolamines in the neuroinflammation: Ultramicronized palmitoylethanolamide in the relief of chronic pain and neurodegenerative diseases. Neuropsychiatry (London).

[131] Nestmann E.R. (2016). Safety of micronized palmitoylethanolamide (microPEA): Lack of toxicity and genotoxic potential. Food Sci. Nutr..

[132] Wise L.E., Cannavacciulo R., Cravatt B.F., Marun B.F., Lichtman A.H. (2008). Evaluation of fatty acid amides in the carrageenan-induced paw edema model. Neuropharmacology.

[133] LoVerme J., Russo R., La Rana G., Fu J., Farthing J., Raso G., Meli R., Hohmann A., Calignano A., Piomelli D. (2006). Rapid broad-spectrum analgesia through activation of peroxisome proliferator-activated receptor-alpha. J. Pharmacol. Exp. Ther..

[134] Alhouayek M., Bottemanne P., Subramanian K.V., Lambert D.M., Makriyannis A., Cani P.D., Muccioli G.G. (2015). N-Acylethanolamine-hydrolyzing acid amidase inhibition increases colon N-palmitoylethanolamine levels and counteracts murine colitis. FASEB J..

[135] Cani P.D., Plovier H., Hul M.V., Geurts L., Delzenne N.M., Druart C., Everard A. (2016). Endocannabinoids-at the crossroads between the gut microbiota and host metabolism. Nat. Rev. Endocrinol..

[136] Capasso R., Matias I., Lutz B., Borrelli F., Capasso F., Marsicano G., Mascolo N., Petrosino S., Monory K., Valenti M. (2005). Fatty acid amide hydrolase controls mouse intestinal motility in vivo. Gastroenterology.

[137] Russo R., Cristiano C., Avagliano C., De Caro C., La Rana G., Raso G.M., Canani R.B., Meli R., Calignano A. (2018). Gut-brain axis: Role of lipids in the regulation of inflammation, pain and CNS diseases. Curr. Med. Chem..

[138] Esposito G., Capoccia E., Turco F., Palumbo I., Lu J., Steardo A., Cuomo R., Sarnelli G., Steardo L. (2014). Palmitoylethanolamide improves colon inflammation through an enteric glia/toll like receptor 4-dependent PPAR-alfa activation. Gut.

[139] Karwad M.A., Macpherson T., Wang B., Theophilidou E., Sarmad S., Barrett D.A., Larvin M., Wright K.L., Lund J.N., O’Sullivan S.E. (2017). Oleoylethanolamine and palmitoylethanolamine modulate intestinal permeability in vitro via TRPV1 and PPARalpha. FASEB J..

[140] Sarnelli G., Seguella L., Pesce M., Lu J., Gigli S., Bruzzese E., Lattanzi R., D’Alessandro A., Cuomo R., Steardo L. (2018). HIV-1 Tat-induced diarrhea is improved by the PPARalpha agonist, palmitoylethanolamide, by suppressing the activation of enteric glia. J. Neuroinflamm..

[141] Pengo G., Miolo A. Utilizzo di Palmitoiletanolamide micronizzata nell’infiammazione gastrointestinale idiopatica (IBD) del cane: Descrizione di 7 casi clinici. Proceedings of the 72 International SCIVAC Congress.

[142] Carta G., Murru E., Vargiu R., Collu M., Carta M., Banni S., Stancampiano R. (2020). Essential fatty acids deficient diet modulates N-Acylethanolamide profile in rat’s tissues. Prostaglandins Leukot. Essent. Fatty Acids..

[143] Borrelli F., Izzo A.A. (2009). Role of acylethanolamides in the gastrointestinal tract with special reference to food intake and energy balance. Best Pract. Res. Clin. Endocrinol. Metab..

[144] Borrelli F., Romano B., Petrosino S., Pagano E., Capasso R., Coppola D., Battista G., Orlando P., Izzo A. (2015). Palmitoylethanolamide, a naturally-occurring lipid, is an orally effective intestinal anti-inflammatory agent. Br. J. Pharmacol..

[145] Capasso R., Orlando P., Pagano E., Aveta T., Buono L., Borrelli F., Di Marzo V., Izzo A.A. (2014). Palmitoylethanolamide normalizes intestinal motility in a model of post-inflammatory accelerated transit: Involvement of CB1 receptors and TRPV1 channels. Br. J. Pharmacol..

[146] Couch D.G., Cook H., Ortori C., Barrett D., Lund J.N., O’Sullivan S.E. (2019). Palmitoylethanolamide and cannabidiol prevent inflammation-induced hyperpermeability of the human gut in vitro and in vivo-a randomized, placebo-controlled, double-blind controlled trial. Inflamm. Bowel Dis..

[147] Di Paola R., Impellizzeri D., Torre A., Mazzon E., Cappellani A., Faggio C., Esposito E., Trischitta F., Cuzzocrea S. (2012). Effects of palmitoylethanolamide on intestinal injury and inflammation caused by ischemia-reperfusion in mice. J. Leukoc. Biol..

[148] Hasenoehrl C., Storr M., Schicho R. (2017). Cannabinoids for treating inflammatory bowel diseases: Where are we and where do we go?. Expert. Rev. Gastroenterol. Hepatol..

[149] Karwad M.A., Couch D.G., Wright K.L., Tufarelli C., Larvin M., Lund J., O’Sullivan S.E. (2019). Endocannabinoids and endocannabinoid-like compounds modulate hypoxia-induced permeability in CaCo-2 cells via CB1, TRPV1, and PPARa. Biochem. Pharmacol..

[150] Cristiano C., Pirozzi C., Coretti L., Cavaliere G., Lama A., Russo R., Lembo F., Mollica M.P., Meli R., Calignano A. (2018). Palmitoylethanolamide counteracts autistic-like behaviours in BTBR T+tf/J mice: Contribution of central and peripheral mechanisms. Brain Behav. Immun..

[151] Pesce M., D’Alessandro A., Borrelli O., Gigli S., Seguella L., Cuomo R., Esposito G., Sarnelli G. (2018). Endocannabinoid-related compounds in gastrointestinal diseases. J. Cell Mol. Med..

[152] Pesce M., Esposito G., Sarnelli G. (2018). Endocannabinoids in the treatment of gastrointestinal inflammation and symptoms. Curr. Opin. Pharmacol..

[153] Sarnelli G., Gigli S., Capoccia E., Iuvone T., Cirillo C., Seguella L., Nobile N., D’Alessandro A., Pesce M., Steardo L. (2016). Palmitoylethanolamide exerts antiproliferative effect and downregulates VEGF signaling in Caco-2 human colon carcinoma cell line through a selective PPAR-a-dependent inhibition of Akt/mTOR pathway. Phytother. Res..

[154] Wang J., Zheng J., Kulkarni A., Wang W., Garg S., Prather P.L., Hauer-Jensen M. (2014). Palmitoylethanolamide regulates development of intestinal radiation injury in a mast cell-dependent manner. Dig. Dis. Sci..

[155] Cremon C., Stanghellini V., Barbaro M.R., Cogliandro R.F., Bellacosa L., Santos J., Vicario M., Pigrau M., Cotoner C.A., Lobo B. (2017). Randomised clinical trial: The analgesic properties of dietary supplementation with palmitoylethanolamide and polydatin in irritable bowel syndrome. Aliment Pharmacol. Ther..

[156] Muccioli G.G., Naslain D., Backhed F., Reigstad C.S., Lambert D.M., Delzenne N.M., Cani P.D. (2010). The endocannabinoid system links gut microbiota to adipogenesis. Mol. Syst. Biol..

[157] Geurts L., Everard A., Van Hul M., Essaghir A., Duparc T., Matamoros S., Plovier H., Castel J., Denis R.G., Bergiers M. (2015). Adipose tissue NAPE-PLD controls fat mass development by altering the browning process and gut microbiota. Nat. Commun..

[158] Guida F., Boccella S., Belardo C., Iannotta M., Piscitelli F., De Filippis F., Paino S., Ricciardi F., Siniscalco D., Marabese I. (2020). Altered gut microbiota and endocannabinoid system tone in vitamin D deficiency-mediated chronic pain. Brain Behav. Immun..

[159] Di Paola M., Bonechi E., Provensi G., Costa A., Clarke G., Ballerini C., De Filippo C., Passani M.B. (2018). Oleoylethanolamide treatment affects gut microbiota composition and the expression of intestinal cytokines in Peyer’s patches of mice. Sci. Rep..

[160] Fornelos N., Franzosa E.A., Bishai J., Annand J.W., Oka A., Lloyd-Price J., Arthur T.D., Garner A., Avila-Pacheco J., Haiser H.J. (2020). Growth effects of N-acylethanolamines on gut bacteria reflect altered bacterial abundances in inflammatory bowel disease. Nat. Microbiol..

[161] Barutta F., Bruno G., Mastrocola R., Bellini S., Gruden G. (2018). The role of cannabinoid signaling in acute and chronic kidney diseases. Kidney Int..

[162] Izzo A.A., Muccioli G.G., Ruggieri M.R., Schicho R. (2015). Endocannabinoids and the digestive tract and bladder in health and disease. Handb. Exp. Pharmacol..

[163] Merriam F.V., Wang Z.Y., Guerios S.D., Bjorling D.E. (2008). Cannabinoid receptor 2 is increased in acutely and chronically inflamed bladder of rats. Neurosci. Lett..

[164] Petrosino S., Puigdemont A., della Valle M.F., Fusco M., Verde R., Allarà M., Orlando P., Aveta T., Di Marzo V. (2016). Adelmidrol increases the endogenous concentrations of palmitoylethanolamide in canine keratinocytes and down-regulates an inflammatory reaction in an in vitro model of contact allergic dermatitis. Vet. J..

[165] Barutta F., Piscitelli F., Pinach S., Bruno G., Gambino R., Rastaldi M.P., Salvidio G., Di Marzo V., Cavallo Perin P., Gruden G. (2011). Protective role of cannabinoid receptor type 2 in a mouse model of diabetic nephropathy. Diabetes.

[166] Zoja C., Locatelli M., Corna D., Villa S., Rottoli D., Nava V., Verde R., Piscitelli F., Di Marzo V., Fingerle J. (2016). Therapy with a selective cannabinoid receptor type 2 agonist limits albuminuria and renal injury in mice with type 2 diabetic nephropathy. Nephron.

[167] Sampaio L.S., Iannotti F.A., Veneziani L., Borelli-Tôrres R.T., De Maio F., Piscitelli F., Reis R.A.M., Di Marzo V., Einicker-Lamas M. (2018). Experimental ischemia/reperfusion model impairs endocannabinoid signaling and Na+/K+ ATPase expression and activity in kidney proximal tubule cells. Biochem. Pharmacol..

[168] Di Paola R., Impellizzeri D., Mondello P., Velardi E., Aloisi C., Cappellani A., Esposito E., Cuzzocrea S. (2012). Palmitoylethanolamide reduces early renal dysfunction and injury caused by experimental ischemia and reperfusion in mice. Shock.

[169] Godlewski G., Offertáler L., Wagner J.A., Kunos G. (2009). Receptors for acylethanolamides-GPR55 and GPR119. Prostaglandins Other Lipid Mediat..

[170] Impellizzeri D., Esposito E., Attley J., Cuzzocrea S. (2014). Targeting inflammation: New therapeutic approaches in Chronic Kidney Disease (CKD). Pharmacol. Res..

[171] Smart D., Jonsson K.O., Vandevoorde S., Lambert D.M., Fowler C.J. (2002). ‘Entourage’ effects of N-acyl ethanolamines at human vanilloid receptors. Comparison of effects upon anandamide-induced vanilloid receptor activation and upon anandamide metabolism. Br. J. Pharmacol..

[172] Cordaro M., Impellizzeri D., Bruschetta G., Siracusa R., Crupi R., Di Paola R., Esposito E., Cuzzocrea S. (2016). A novel protective formulation of Palmitoylethanolamide in experimental model of contrast agent induced nephropathy. Toxicol. Lett..

[173] Mattace Raso G., Simeoli R., Russo R., Santoro A., d’Emmanuele di Villa Bianca R., Mitidieri E., Paciello O., Orefice N., Meli R., Calignano A. (2013). N-palmitoylethanolamide protects the kidney from hypertensive injury in spontaneously hypertensive rats via inhibition of oxidative stress. Pharmacol. Res..

[174] Impellizzeri D., Bruschetta G., Ahmad A., Crupi R., Siracusa R., Di Paola R., Paterniti I., Prosdocimi M., Esposito E., Cuzzocrea S. (2015). Effects of Palmitoylethanolamide and silymarin combination treatment in an animal model of kidney ischemia and reperfusion. Eur. J. Pharmacol..

[175] Merriam F.V., Wang Z.Y., Hillard C.J., Stuhr K.L., Bjorling D.E. (2011). Inhibition of fatty acid amide hydrolase suppresses referred hyperalgesia induced by bladder inflammation. BJU Int..

[176] Pessina F., Capasso R., Borrelli F., Aveta T., Buono L., Valacchi G., Fiorenzani P., Di Marzo V., Orlando P., Izzo A.A. (2015). Protective effect of palmitoylethanolamide, a naturally-occurring molecule, in a rat model of cystitis. J. Urol..

[177] Farquhar-Smith W.P., Jaggar S.I., Rice A.S. (2002). Attenuation of nerve growth factor-induced visceral hyperalgesia via cannabinoid CB(1) and CB(2)-like receptors. Pain.

[178] Jaggar S.I., Hasnie F.S., Sellaturay S., Rice A.S. (1998). The anti-hyperalgesic actions of the cannabinoid anandamide and the putative CB2 receptor agonist palmitoylethanolamide in visceral and somatic inflammatory pain. Pain.

[179] Farquhar-Smith W.P., Rice A.S. (2003). A novel neuroimmune mechanism in cannabinoid-mediated attenuation of nerve growth factor-induced hyperalgesia. Anesthesiology.

[180] Farquhar-Smith W.P., Rice A.S. (2001). Administration of endocannabinoids prevents a referred hyperalgesia associated with inflammation of the urinary bladder. Anesthesiology.

[181] Petrini D., Di Giuseppe M., Deli G., De Caro Carella C. (2016). Cystolithiasis in a Syrian hamster: A different outcome. Open Vet. J..

[182] Lamont L.A. (2008). Multimodal pain management in veterinary medicine: The physiologic basis of pharmacologic therapies. Vet. Clin. North Am. Small Anim. Pract..

[183] Calignano A., La Rana G., Giuffrida A., Piomelli D. (1998). Control of pain initiation by endogenous cannabinoids. Nature.

[184] Piomelli D., Sasso O. (2014). Peripheral gating of pain signals by endogenous lipid mediators. Nat. Neurosci..

[185] Koltyn K.F., Brellenthin A.G., Cook D.B., Sehgal N., Hillard C. (2014). Mechanisms of exercise-induced hypoalgesia. J. Pain.

[186] Paladini A., Fusco M., Cenacchi T., Schievano C., Piroli A., Varrassi G. (2016). Palmitoylethanolamide, a special food for medical purposes, in the treatment of chronic pain: A pooled data meta-analysis. Pain Phys..

[187] Cruccu G., Di Stefano G., Marchettini P., Truini A. (2019). Micronized palmitoylethanolamide: A post-hoc analysis of a controlled study in over 600 patients with low back pain-sciatica. CNS Neurol. Disord. Drug Targets.

[188] De Filippis D., Luongo L., Cipriano M., Palazzo E., Cinelli M.P., de Novellis V., Maione S., Iuvone T. (2011). Palmitoylethanolamide reduces granuloma-induced hyperalgesia by modulation of mast cell activation in rats. Mol. Pain.

[189] Esposito E., Paterniti I., Mazzon E., Genovese T., Di Paola R., Galuppo M., Cuzzocrea S. (2011). Effects of palmitoylethanolamide on release of mast cell peptidases and neurotrophic factors after spinal cord injury. Brain Behav. Immun..

[190] Genovese T., Esposito E., Mazzon E., Di Paola R., Meli R., Bramanti P., Piomelli D., Calignano A., Cuzzocrea S. (2008). Effects of palmitoylethanolamide on signaling pathways implicated in the development of spinal cord injury. J. Pharmacol. Exp. Ther..

[191] Skaper S.D., Facci L., Giusti P. (2013). Glia and mast cells as targets for palmitoylethanolamide, an anti-inflammatory and neuroprotective lipid mediator. Mol. Neurobiol..

[192] Britti D., Crupi R., Impellizzeri D., Gugliandolo E., Fusco R., Schievano C., Morittu V.M., Evangelista M., Di Paola R., Cuzzocrea S. (2017). A novel composite formulation of palmitoylethanolamide and quercetin decreases inflammation and relieves pain in inflammatory and osteoarthritic pain models. BMC Vet. Res..

[193] Vezzoni A., Crupi F., Boiocchi S., Boano S. Effect of palmitoylethanolamide co-ultra micronized with quercetin in dogs with osteoarthritis by means of dynamic gate analysis and canine brief pain inventory questionnaire. Proceedings of the 5th World Veterinary Orthopaedic Congress ESVOT-VOS.

[194] Neugebauer V., Han J.S., Adwanikar H., Fu Y., Ji G. (2007). Techniques for assessing knee joint pain in arthritis. Mol. Pain.

[195] Orita S., Ishikawa T., Miyagi M., Ochiai N., Inoue G., Eguchi Y., Kamoda H., Arai G., Toyone T., Aoki Y. (2011). Pain-related sensory innervation in monoiodoacetate-induced osteoarthritis in rat knees that gradually develops neuronal injury in addition to inflammatory pain. BMC Musculoskelet. Disord..

[196] Della Valle M.F., Mortellaro C.M., Miolo A., Costa B. Aliamides for pain management of osteoarthritis. Proceedings of the 3rd Vepra Conference.

[197] Cordaro M., Siracusa R., Impellizzeri D., D’Amico R., Peritore A.F., Crupi R., Gugliandolo E., Fusco R., Di Paola R., Schievano C. (2019). Safety and efficacy of a new micronized formulation of the ALIAmide palmitoylglucosamine in preclinical models of inflammation and osteoarthritis pain. Arthritis Res. Ther..

[198] Miolo A., della Valle M.F., Impellizzeri D., Siracusa R., Cordaro M., Di Paola R., Cuzzocrea S. Micronized palmitoyl-glucosamine, alone or co-micronized with curcumin, decreases inflammation, chondrodegeneration and pain: A preclinical study. Proceedings of the 5th World Veterinary Orthopaedic Congress ESVOT-VOS.

[199] De Filippis D., D’Amico A., Cinelli M.P., Esposito G., Di Marzo V., Iuvone T. (2009). Adelmidrol, a palmitoylethanolamide analogue, reduces chronic inflammation in a carrageenin-granuloma model in rats. J. Cell Mol. Med..

[200] Impellizzeri D., Di Paola R., Cordaro M., Gugliandolo E., Casili G., Morittu V.M., Britti D., Esposito E., Cuzzocrea S. (2016). Adelmidrol, a palmitoylethanolamide analogue, as a new pharmacological treatment for the management of acute and chronic inflammation. Biochem. Pharmacol..

[201] Abramo F., Salluzzi D., Leotta R., Auxilia S., Noli C., Miolo A., Mantis P., Lloyd D.H. (2008). Mast cell morphometry and densitometry in experimental skin wounds treated with a gel containing adelmidrol: A placebo controlled study. Wounds.

[202] Mantis P., Lloyd D.H., Pfeiffer D., Stevens K., Auxilia S., Noli C., Abramo F., Miolo A. (2007). Assessment of the effect of an aliamide-containing topical gel by evaluation of the reduction of wound volume measured by high resolution ultrasound biomicroscopy. Wounds.

[203] Siracusa R., Impellizzeri D., Cordaro M., Gugliandolo E., Peritore A.F., Di Paola R., Cuzzocrea S. (2018). Topical application of adelmidrol + trans-traumatic acid enhances skin wound healing in a streptozotocin-induced diabetic mouse model. Front. Pharmacol..

[204] Pulvirenti N., Nasca M.R., Micali G. (2007). Topical adelmidrol 2% emulsion, a novel aliamide, in the treatment of mild atopic dermatitis in pediatric subjects: A pilot study. Acta Dermatovenerol. Croat..

[205] Karatzi C., Stefanidou M., Chaniotis V., Evangelou G., Krueger-Krasagakis S., Krasagakis K. (2018). Treatment of giant vulvar syringomas with topical adelmidrol: The role of mast cells. Australas. J. Dermatol..

[206] Cerrato S., Brazis P., della Valle M.F., Miolo A., Puigdemont A. (2012). Inhibitory effect of topical Adelmidrol on antigen-induced skin wheal and mast cell behavior in a canine model of allergic dermatitis. BMC Vet. Res..

[207] Fabbrini F., Leone F. (2013). Topical adelmidrol (2%) in the management of pruritus associated with atopic dermatitis in dogs-An observational study. Veterinaria.

[208] Bonello D., Squarzoni P. (2008). Effect of a mucoadhesive gel and dental scaling on gingivitis in dogs. J. Vet. Dent..

[209] Zerweck C., Grove G., Fraser J. (2006). Efficacy of S236 cream in promoting barrier repair of razor-induced skin trauma. J. Am. Acad. Dermatol..

[210] Vaia M., Petrosino S., De Filippis D., Negro L., Guarino A., Carnuccio R., Di Marzo V., Iuvone T. (2016). Palmitoylethanolamide reduces inflammation and itch in a mouse model of contact allergic dermatitis. Eur. J. Pharmacol..

[211] Marsella R., Joyce J., Nicklin C., Lopez J. (2005). Evaluation of the effects of Palmitoylethanolamide on clinical signs in house dust mite allergic high IgE Beagle dogs using a randomized, double blinded, placebo controlled design. Vet. Dermatol..

[212] Noli C., della Valle M.F., Miolo A., Medori C., Schievano C., Skinalia Clinical Group (2015). Efficacy of ultra-micronized palmitoylethanolamide in canine atopic dermatitis: An open-label multi-centre study. Vet. Dermatol..

[213] Noli C., della Valle M.F., Miolo A., Medori C., Schievano C., Skinalia Clinical Group (2019). Effect of dietary supplementation with ultramicronized palmitoylethanolamide in maintaining remission in cats with nonflea hypersensitivity dermatitis: A double blind, multicentre, randomized, placebo-controlled study. Vet. Dermatol..

